# Debottlenecking Thermophilic Cyanobacteria Cultivation and Harvesting through the Application of Inner-Light Photobioreactor and Chitosan

**DOI:** 10.3390/plants10081540

**Published:** 2021-07-27

**Authors:** Hairuo Zhang, Pengyu Chen, Mohammad Russel, Jie Tang, Peng Jin, Maurycy Daroch

**Affiliations:** 1 School of Environment and Energy, Peking University Shenzhen Graduate School, 2199 Lishui Rd., Shenzhen 518055, China; 13164245035@163.com (H.Z.); 1801214499@pku.edu.cn (P.C.); jinpeng@pkusz.edu.cn (P.J.); 2 School of Ocean Science and Technology, Dalian University of Technology, Panjin 124221, China; mrussel@dlut.edu.cn; 3 School of Food and Bioengineering, Chengdu University, Chengdu 610052, China; tangjie@cdu.edu.cn

**Keywords:** thermophilic cyanobacteria, bioreactor, light attenuation, chitosan, flocculation

## Abstract

Thermophilic cyanobacteria are a low-carbon environmental resource with high potential thanks to their innate temperature tolerance and thermostable pigment, phycocyanin, which enhances light utilisation efficiency and generates a high-value product. However, large-scale cultivation and harvesting have always been bottlenecks in unicellular cyanobacteria cultivation due to their micrometric size. In this study, a 40-litre inner-light photobioreactor (PBR) was designed for scaled-up cultivation of *Thermosynechococcus elongatus* E542. By analysing light transmission and attenuation in the PBR and describing it via mathematical models, the supply of light energy to the reactor was optimised. It was found that the hyperbolic model describes the light attenuation characteristics of the cyanobacterial culture more accurately than the Lambert–Beer model. The internal illumination mode was applied for strain cultivation and showed a two-fold better growth rate and four-fold higher biomass concentration than the same strain grown in an externally illuminated photobioreactor. Finally, the downstream harvesting process was explored. A mixture of chitosan solutions was used as a flocculant to facilitate biomass collection. The effect of the following parameters on biomass harvesting was analysed: solution concentration, flocculation time and flocculant concentration. The analysis revealed that a 4 mg L^−1^ chitosan solution is optimal for harvesting the strain. The proposed solutions can improve large-scale cyanobacterial biomass cultivation and processing.

## 1. Introduction

In recent years, research in cyanobacterial biotechnology achieved a lot of interest, but their application capacity cannot achieve its potential due to the low efficiency of large-scale culture and the downstream harvesting process. Cyanobacteria are natural producers of valuable compounds such as phycobiliproteins [1], biopolymers [2] and speciality chemicals [3] and are useful candidates for the bioremediation of water [4]. It appears, however, that genetic engineering and carbon dioxide valorisation are the most promising disciplines where cyanobacteria can make a long-lasting difference, especially after the discovery and development of new faster growing and more robust strains [5,6,7]. To date, important platform chemicals such as isoprene, ethylene, alkanes, etc. have been produced in cyanobacteria [3,8]. To fully explore the industrial potential of these organisms, thorough studies are needed on the factors that currently limit their industrial application. Two crucial areas where such improvements are needed are light energy transfer and harvesting of the biomass, both significant bottlenecks of cyanobacterial biotechnology.

Photobioreactors (PBR) are critical equipment required for the efficient cultivation of photosynthetic microorganisms. Therefore, improving PBR efficiency is crucial for the industrialisation of cyanobacterial technology. The optimisation of light energy distribution and light energy transfer within the PBR can improve the productivity of culture systems. Understanding light attenuation through modelling is a promising way to optimise the light energy distribution to achieve the improvement of PBR efficiency. In principle, light attenuation should be homogeneous to obtain good growth parameters. However, irradiance decreases exponentially with an increasing volume of the culture, and obtaining homogenous irradiance in the whole bioreactor system is challenging.

Two methods can be applied to enhance the light availability and decrease attenuation: develop a rapid cell mixing system or employ a light guide material. A combination of both approaches can also be used. Since most bioreactors use external lighting, it is very problematic to achieve uniform illumination of the cell suspension, especially at high biomass densities. Therefore, several approaches to tackle such issues have been used. Jung et al. [9] used 3D printing technology to produce a bioreactor with multiple layers of rough slides embedded in it. Light penetrates along the edge of the slide and is refracted by the slide to guide the light to the bacteria. Hsieh et al. [10] placed a transparent plexiglass cuboid at the bottom of the reactor, with light emanating from the bottom of the cuboid and around it. This resulted in a sufficient illumination of the cells at the bottom of the reactor and a 56% increase in *Chlorella* production. Ahsan et al. [11] improved a photosynthetic microbial photobioreactor with a built-in V-groove optical panel machined from transparent plexiglass, in which light is shone through the top of the reactor into a V-shaped trough. It is then transmitted laterally to the bacterial cells, which also improves the biomass yield. Chen et al. [12] used a Fresnel lens to gather sunlight and then directed the gathered sunlight into an optical fibre, where the light was transmitted into the reactor. Xue et al. [13] also used the same method of converging sunlight and introducing it into optical fibres for transmission to the reactor and enhanced the flash effect to increase the biomass of the algae species by more than 40%.

Another method of enhancing light transfer is to add a spoiler or an aeration device to the inside of the photobioreactor. This approach speeds up the flow of cells in the reactor and facilitates their movement between light and dark zones, triggering the flash effect and accelerating the accumulation of cellular biomass. Huang et al. [14] placed multiple inclined inserts on the inside of the reactor and simulated their function with computational fluid dynamics software. Degen et al. [15] added a baffle plate to the reactor and divided it into two parts, the ascending zone and the descending zone, and placed an aeration device at the bottom of the ascending zone. Photosynthetic microorganisms were continually transferred between ascending and descending zones and zones of light and dark. Wang et al. [16] improved the bioreactor by retaining only the rising zone and adding a more convenient horizontal baffle to obtain higher biomass yields. Yang et al. [17] built several cylinders and prisms into the plate photobioreactor, which increased the internal turbulence, mixing and exposure to light. This approach has increased the yield of algal biomass by more than 70% due to the good attenuation effect compared with the control group without the perturbation device.

Cyanobacteria have numerous advantages during cultivation and with regards to their photosynthetic apparatus due to the presence of phycobiliproteins, which can utilise a larger spectrum of light than most green algae by expanding it to the range not accessible for chlorophyll [1,18]. The maximal light fluxes of the rapidly-growing strains of cyanobacteria such as *Synechococcus elongatus* UTEX 2973 allow some of them to use highly concentrated light at the 1500 μmol m^−2^ s^−1^ level, which is unsuitable for most green algae [19]. Cyanobacteria culture density is highly dependent on environmental conditions. The key factors controlling population density are the availability of light and carbon. Since most or sometimes all of the reducing equivalents, ATP and NADPH, that are necessary for carbon fixation in cyanobacteria are derived from light reactions of photosynthesis, the supply of light has a major impact on the efficiency of carbon fixation. In this way, the growth rate of cyanobacteria is directly controlled by gross photosynthetic rates and related to photo-irradiation [20]. In photobioreactor cultivation, the light supply is the primary constraint of cyanobacterial growth, with carbon supply being the secondary one. The optimal light supply will have a knock-on effect on downstream metabolic activities and the production of valuable metabolites. Conversely, oversaturation of the culture with light is deleterious to its vitality due to the effect known as photoinhibition, formation of light reaction-derived reactive oxygen species and photobleaching that limits the productivity of photosynthetic cells, especially at low cellular densities [21]. The growth of a photosynthetic organism in a dense culture is a dynamic process. Initially, excessive light is a major problem generating cellular stress. However, with the rise of cellular density and increasing effects of shading and scattering, light limitation becomes a significant problem. These effects are more profound in large scale bioreactors where optical paths are longer and light attenuation effects of both cells and culture medium are more significant.

The third constraint of cyanobacterial biomass cultivation after light and carbon supply is biomass harvesting. Due to the small cell size of many of these unicellular prokaryotes, their harvesting is even more complex than that of eukaryotic microalgae. Due to the small, micrometric size of cyanobacteria and cell walls, which typically lack cellulose, they are easier to break compared to eukaryotic green algae. The negatively-charged cells disperse uniformly in the culture medium, bringing specific difficulties during their separation and harvesting. One promising method to tackle the problem is the utilisation of a cationic polymer, chitosan [22]. Chitosan, which contains positively charged groups, can act as an ionic bridge between negatively-charged cyanobacterial cells. Reversibility and ionic strength-dependence of this mechanism is an added advantage that protects the high-value products from degradation during harvesting. Beach et al. [23] tested two methods of green algae harvesting methods: Al-Fe metal salt and chitosan. Their results showed that the flocculation efficiency of chitosan was as high as 95%, and the harvesting efficiency was better than that of metal salt. Vu et al. [24] found that ferric chloride was more effective than chitosan in flocculating *Chlorella* sp. On the other hand, their study suggested that chitosan had better flocculation efficiency than metal salts. Chitosan has shown a synergistic effect with metal salts, wherein the flocculation efficiency exceeded 80%, which is significantly higher than that of a single flocculant.

In recent years we have developed a strain of thermophilic unicellular cyanobacteria of a hot-spring abundant genus *Thermosynechococcus* [25] into a promising thermophilic photosynthetic cell factory for CO_2_ utilisation [5]. One of the biggest challenges in using this strain as a cell factory was to develop an efficient cultivation and harvesting method that could be employed in a large scale that allows sufficient amounts of biomass for the production of valuable compounds such as phycocyanins.

In this study, we addressed these bottlenecks of the process by proposing a new, improved design of photobioreactor based on light attenuation studies to control the light distribution inside the bioreactor and fine-tune it to culture requirements. We have also shown that small concentrations of chitosan are an optimal solution for efficient harvesting of micrometric-size cyanobacteria. All these findings will help to advance the wider deployment of thermophilic cyanobacteria.

## 2. Results

### 2.1. Light Attenuation Curve and Light Exposure Characteristics of Thermosynechococcus E542

Light is indispensable for all photosynthetic microorganisms. Its efficient delivery to the cyanobacterial culture is of primary importance for the effective cultivation of these photosynthetic microbes. Since each of the cyanobacterial cells interacts with light using mechanisms of both absorption and scattering in the presence of interference of the growth medium, this results in incident light attenuation that is a function of the cellular density. It is therefore of primary importance to understand the compound effect of light attenuation in cyanobacterial cultures.

**Figure 1:**
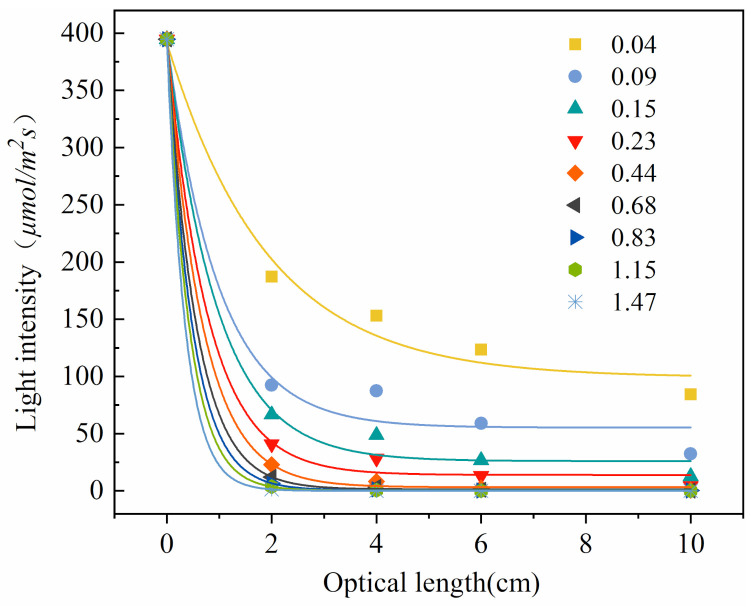
Light attenuation curve of cyanobacteria E542 at various OD.

The light attenuation relationship is described as a function of light intensity, wavelength and optical density of cells. *Thermosynechococcus* E542 cells are in the logarithmic phase of growth when the optical density is 0.6–1.0 (Equations (1) and (2)). Their growth trend gradually stabilises when the optical density value reaches 1.5. This study found that cyanobacteria E542 biomass growth is directly dependent on light and limited at light intensity 0.9084 µmol m^−2^ s^−1^; at *OD*_685nm_ 0.68, at 6 cm optical length range with the incident light intensity of 400 μmol m^−2^ s^−1^ as shown in Figure 1. This means that in medium to large PBRs, a large proportion of photosynthetic microorganisms are not adequately illuminated during the logarithmic growth phase, thus resulting in growth inhibition due to photosynthetic energy shortage. When biomass optical density reaches 1.47, the light intensity drops to 1.333 µmol m^−2^ s^−1^ at an optical length of 2 cm. It is important to mention that the optical length of 2 cm is comparable to most laboratory culture vessels. To summarise, light availability appears to be the major constrain for the growth of cyanobacteria in high-density cultures.

OriginPro2018 was used to fit the light attenuation data within the heat-resistant cyanobacteria E542 culture system, and the fitted model used the first-order attenuation exponential function (ExpDec1) to obtain the light attenuation curves at nine concentrations as shown in Figure 1. The fitting formula and correlation test of light attenuation are shown in Table 1.

**Figure 2:**
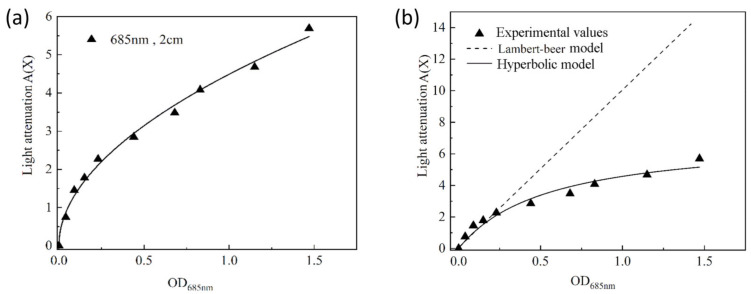
Comparison of the light attenuation coefficient *A(X)* predicted by the Lambert–Beer model and the hyperbolic model with the experimental data. (**a**) The best fit logistic model with experimental values; (**b**) Fitting of experimental values to the Lambert–Beer and hyperbolic models.

The optical attenuation coefficients *A(X)* at different optical densities were fitted linearly to obtain the trend of optical attenuation coefficients with biomass optical density via Lambert–Beer’s model (Equation (3)), as shown in Figure 2a. It can be seen that biomass concentration and the light attenuation relationship of heat-resistant cyanobacterium E542 is not consistent with Lambert–Beer’s law. The curve shows that the optical attenuation coefficient increases linearly with an optical density only at very low concentrations (approximately *OD*_685nm_ of 0.1). Since the number of cells in the system is small, the bacterial cells are uniformly exposed to the light, and the mutual shading between cells is not apparent. When the concentration of biomass is increasing, the light attenuation coefficient no longer shows a linear relationship with OD. When scattering and refraction between the cells and the cells’ own light absorption seriously affect the distribution of light in the system, the light attenuation increases. It can be concluded that Lambert–Beer’s law is not appropriate in describing the light attenuation in the culture system of the cyanobacteria.

Attempts were made to identify the mathematical model that better describes the relationship between optical density and light attenuation. The best match between the actual measurements was found for the hyperbolic model (Figure 2b). A nonlinear fit of the optical attenuation coefficients *A(X)* and *OD*_685nm_ was performed in OriginPro2018 using the hyperbolic model (Equation (4)) to obtain the trend of optical attenuation coefficients with biomass optical density. The data fitting was consistent with the hyperbolic model (Table 1), as shown in Figure 2b. A nonlinear fit of the optical attenuation coefficients *A(X)* and *OD*_685nm_ was performed in OriginPro2018 using the Hyperbolic model to obtain the trend of optical attenuation coefficients with biomass optical density consistent with the hyperbolic model, as shown in Figure 2b. The relationship has been solved at *A*_max_ = 7.0795 ± 0.76598 m^−1^, *K_at_* = 0.54743 ± 0.1408 with *R*^2^ = 0.96 indicating satisfactory description of the data, especially at high cellular concentrations.

**Figure 3:**
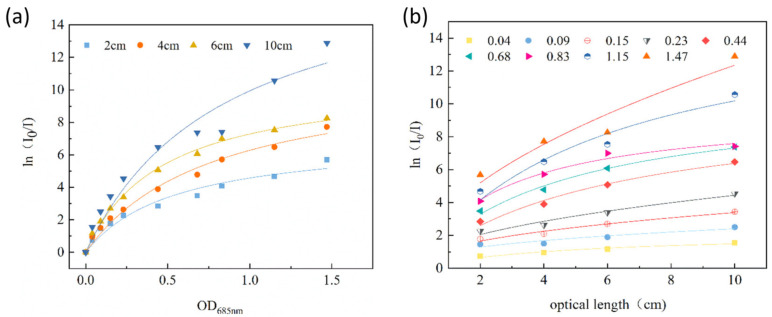
Application of light distribution model with different *OD* and light paths. (**a**) Relationship between optical attenuation coefficient and *OD*, (**b**) Relationship between optical attenuation coefficient and optical length.

To improve the mathematical representation even further, and relate it to the photobioreactor of a specific geometry and light intensity, it was decided that on the basis of the hyperbolic model, a numerical simulation light distribution model should be derived that takes into consideration both light scattering and absorption (Equation (5)). The data, including light incident and transmitted radiation intensities *ln(I*_0_*/I)*, optical length *L* and optical density at *OD*_685nm_, were constructed in Mathematica 12.0. Nonlinear regression analysis was performed to fit the data to the model. The following parameters have been achieved: *P_m_* = 23.5558, *K_xc_* = 4.76169, *K_r_* = 0.672164, *R*^2^ = 0.91, where *P_m_*: maximum light absorption coefficient, *K_xc_*: scattering coefficient of light energy by bacterial cells, and *K_r_*: scattering coefficient of light energy by optical path, respectively (Equations (5) and (6)). The light distribution model was used to assist in the construction and operation of the photobioreactor and to maintain a satisfactory illumination of the culture during the cultivation process. The light distribution model can assist in better understanding light energy transfer in the photobioreactor and optimising light energy delivery (Figure 3). Therefore, the probability of excessive bacterial stress and death and resultant experimental failure can be reduced in the actual operation of the photobioreactor.

### 2.2. Design of the Internal Illumination Photobioreactor and Cultivation of Cyanobacteria

**Figure 4:**
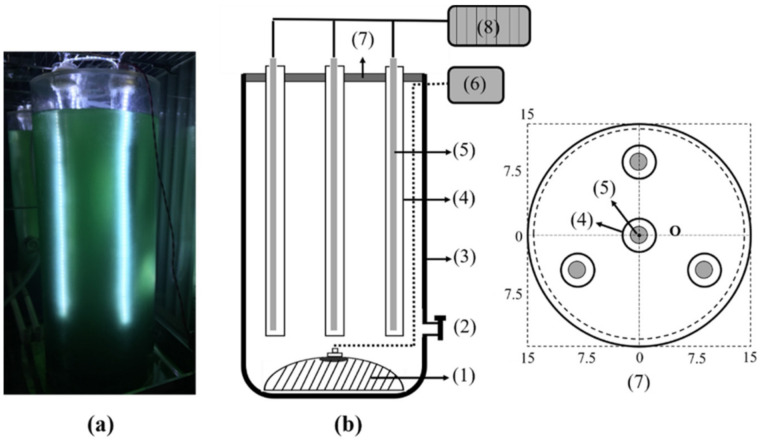
Inner light bioreactor system. (**a**) Picture of PBR. (**b**) Schematic of PBR. (1) Diffuser stone; (2) Harvesting valve; (3) Reactor; (4) Glass tube; (5) LED; (6) air pump; (7) PBR cover; (8) power supply.

An inner-light photobioreactor was built, as shown in Figure 4. Table 2 lists the key parameters derived from the light attenuation study. The culture system consists of three parts: the reactor shell, the light system and the aeration system.

The photobioreactor was utilised for the cultivation of *Thermosynechococcus* E542 in a regime derived from the following assumptions. The light range at different concentrations of *I_s_* = 120 μmol m^−2^ s^−1^ was determined according to the fitting formula (Table 1), from which the light to dark zone ratio was calculated at different *OD*_685nm_ in the reactor as shown in Table 2. The dark zone indicates that the light intensity is still within the acceptable range for the cyanobacterial strain, and the light zone represents the region where photoinhibition is likely to occur. When the incident light intensity is determined as *I*_0_ = 394.67 μmol m^−2^ s^−1^, and with *OD*_685nm_ of cyanobacteria of 0.44, the dark zone in the reactor reached 85%. This indicated most of the bacteria cells could carry out normal photosynthesis within this light intensity. On the other hand, when the bacteria were just inoculated until the *OD*_685nm_ of the reactor was less than 1, the dark zone in the reactor was lower than 80%. Thus, over 20% of the bacteria cells were exposed to excessive light intensity (Table 3). Therefore, at the initial stage of inoculation, the light intensity could be reduced concerning the above-mentioned light-dark partitioning and then gradually increased as needed when the algae grow to an optical density of about 0.5. It could be seen that *I*_0_ = 394.67 μmol m^−2^ s^−1^ can ensure most (85%) of the bacterial cells maintain good lighting conditions, and it could be adjusted within this range according to culture progress to avoid excessive illumination. With the growth and reproduction of bacterial cells, the light intensity can be increased to avoid the light attenuation caused by high *OD*_685nm_.

The above-mentioned principles were utilised to calculate the growth parameters in an initial run of an internally illuminated photobioreactor. For *I*_0_ = 150 μmol m^−2^ s^−1^ the specific growth rate was 0.088 d^−1^, and the value raised to 0.094 d^−1^ for *I*_0_ = 230 μmol m^−2^ s^−1^ at *OD*_685nm_ of 0.15. These values correspond to doubling times of 8.23 d and 7.72 d, respectively. Growth parameters were comparable to the shake-flask cultivations of *Thermosynechococcace* and present no improvement over those methods.

**Figure 5:**
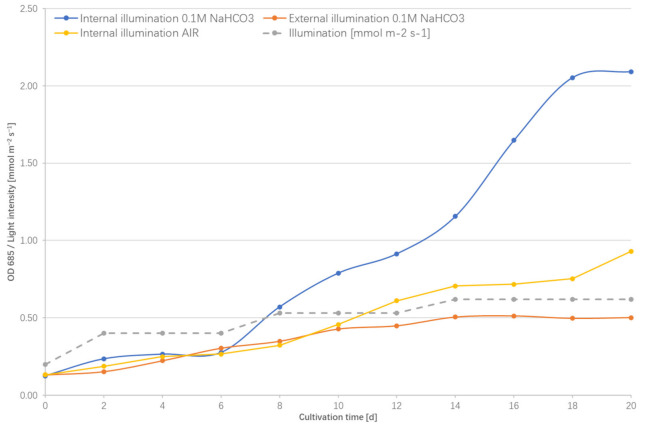
Growth curves of *Thermosynechococcus* E542 grown in following parameters. Internally illuminated photobioreactor with the addition of 0.1 M NaHCO_3_ to the growth medium (blue). Internally illuminated photobioreactor aerated with air (yellow). Externally illuminated photobioreactor with the addition of 0.1 M NaHCO_3_ to the growth medium (orange). The dashed line represents the light gradient of illumination in mmol m^−2^ s^−1^.

On the basis of these initial findings, the strain cultivation regime has been developed to adjust the light intensity to: *OD*_685_ 0.1 to 0.2, 200 μmol m^2^ s^−1^; *OD*_685_ 0.2 to 0.4, 400 μmol m^2^ s^−1^; and *OD*_685_ > 0.4, 530 μmol m^2^ s^−1^. The results of this cultivation strategy are presented below. Simultaneously, the control experiment was performed, in which the equivalent lighting parameters were delivered from the outside of the photobioreactor. The results of these cultivations are shown in Figure 5. Analysis of the growth curves of the strain cultivated in the internally illuminated photobioreactor against the same strain grown in the equivalent external lighting suggests that both methods of cultivation are comparable only at very low *OD*_685_ (cellular densities) that approach 0.3 and illuminations not higher than 400 μmol m^2^ s^−1^. Above these parameters, the density of cyanobacterial culture and associated light scattering effects effectively limit the light penetration inside the column, and the external illumination approach becomes ineffective. In comparison, the internal illumination approach is free of such limitations, allowed for four-fold higher cell densities and achieved maximal specific growth rate during the exponential phase of 0.16 d^−1^, compared to 0.10 d^−1^ of the strain cultivated in the externally illuminated bioreactor. Moreover, the analysis of the trend between illumination parameters and growth curve suggests that a step-wise increase of the illumination parameters has a very positive effect on the growth of the strain by on the one hand, preventing photoinhibition and, on the other hand, providing optimal delivery of light energy for metabolism.

The analysis of the effect of an externally-added bicarbonate source on the growth of the strain in the bioreactor shows that the carbon delivery method also has an important effect on the cultivation of the strain. In the bioreactor aerated with air, without the addition of bicarbonate, despite using the internal illumination method, growth parameters are much lower than those of the bicarbonate-enriched culture. The maximal specific growth rate is 0.11 d^−1^, which is similar to the light-limited culture. This suggests that in the case of thermophilic cyanobacteria, utilisation of air as a carbon dioxide source is suboptimal and is likely to result in carbon limitation. Cyanobacteria uptake different carbon species using different mechanisms; CO_2_ is typically taken up passively and transformed into bicarbonate inside the cell by carbonic anhydrase, an enzyme that maintains the balance between intracellular inorganic carbon species [26]. The alternative route for CO_2_ entry is directly through a bicarbonate ion HCO_3_^-^. There are several mechanisms regarding how HCO_3_^−^ enters the cyanobacterial cell, but in the E542 strain, it is likely to be through *sbtA*, a sodium-dependent bicarbonate transporter A, a high-affinity Na^+^ -dependent HCO_3_^−^ symporter [5,27]. Considering that the solubility of CO_2_ is progressively lower with an increase in temperature and that the majority of cellular inorganic carbon is stored as soluble HCO_3_^−^ and not in its gaseous form, it is bicarbonate that enters the carboxysome before being transformed to CO_2_ for carbon fixation [28]. This is optimal to deliver carbon as bicarbonate to thermophilic cyanobacteria to limit the potential carbon shortage. There are also other practical aspects of using a bicarbonate platform: bicarbonates are readily available, storable for long periods of time in solid and liquid forms and are less expensive to transport than gaseous CO_2_. Finally, the pH of the medium increases as a result of photosynthesis during the consumption of HCO_3_^−^, making the medium more alkaline, which in turn is conducive to capturing more CO_2_ from the air and solubilising it in a growth medium. During the cultivation of the E542 in bicarbonate-enriched medium in an internally illuminated PBR, the pH of the medium raised from an initial 8.65 to 10.30 throughout the 18 days of cultivation.

### 2.3. Chitosan Flocculation Results

**Figure 6:**
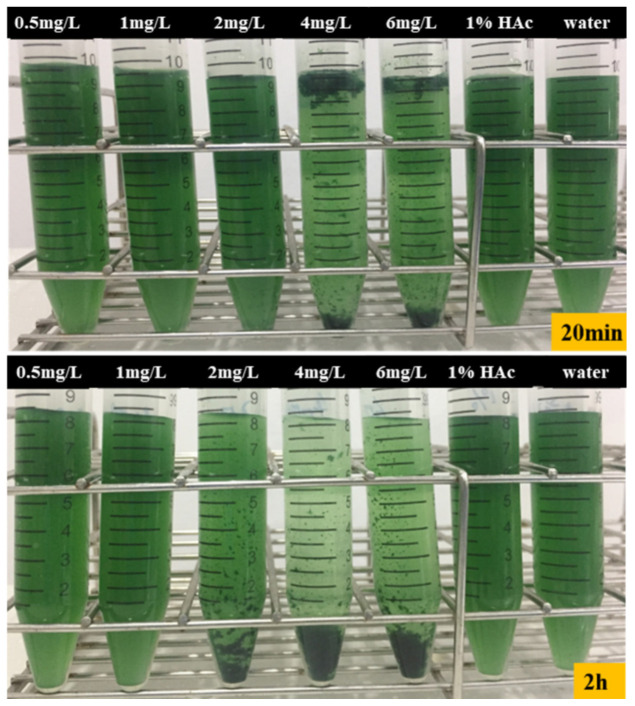
From right to left the concentration of chitosan solution is 0.5, 1, 2, 4, 6 mg L^−1^, 1% acetic acid (HAc) and flocculation after 20 min and 2 h of chitosan.

*Thermosynechococcaceae*, including the E542 strain [29], are typically less than 1 μm in diameter and 2–3 μm in length, sizes typical for picocyanobacteria. This is the main reason why their harvesting is particularly challenging. To tackle this problem, we have employed various concentrations of a natural flocculant, chitosan, to exploit the electrostatic interactions between negatively charged cell walls and this cationic biopolymer. The following final concentrations of chitosan have been tested for their effect on E542 strain harvesting: 0.5, 1, 2, 4 and 6 mg L^−1^. The results have shown that the final concentration of the flocculant between 4 mg L^−1^ to 6 mg L^−1^ proved to be effective in cell harvesting during 2 h of treatment. Meanwhile, in a control experiment where 1% glacial acetic acid was used, no cell flocculation was observed. There was a positive correlation observed between the concentration of chitosan used and the number of flocculated cells. In the range of 0.5–1 mg L^−1^ concentration of the flocculant, the cell clustering potential was negligible and comparable to gravity sedimentation. On the other hand, a positive effect was observed when the concentration of flocculant was 2 mg L^−1^. The flocculation efficiency of 67% was calculated, and was linear within a short period of time, 2 h. The optimal flocculation efficiency of approximately 90% was obtained for the chitosan concentration 4–6 mg L^−1^. This flocculation process could be divided into two phases (Figure 6); in the first 20 min, initial flocks were formed rapidly, and 70% of the cells were flocculated and clustered together at the surface of the medium (Figure 6). In the second phase, these clusters settled at the bottom of the tube, ultimately reaching 90% efficiency. The subsequent second phase of the process is much slower and most likely due to relatively low residual cell count. This phenomenon could be explored as a two-stage harvesting method where, in the initial stages, the floated cells are skimmed from the top of the culture medium and subsequently removed from the settling component of the vessel, or photobioreactor.

**Figure 7:**
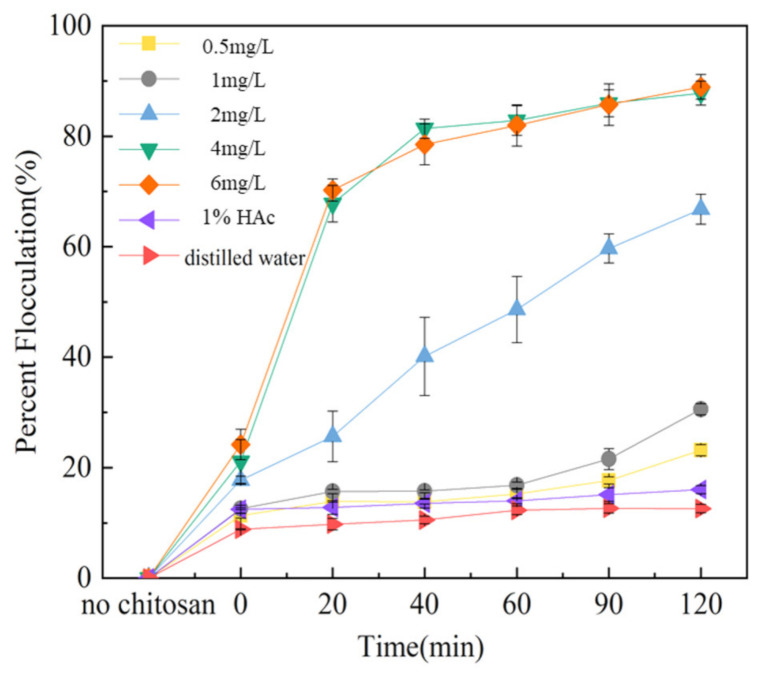
Flocculation efficiency over time of chitosan group.

**Figure 8:**
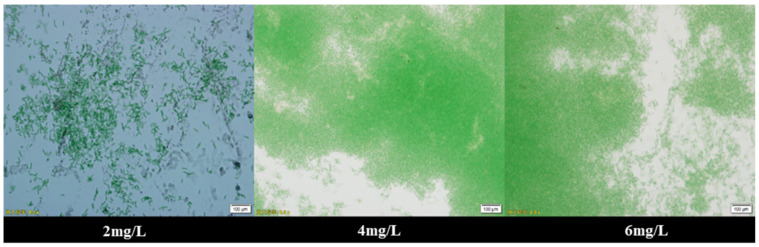
The flocculated cyanobacteria form at 40 times magnification after 2 h of flocculation with different concentrations of chitosan. The concentration of chitosan solution from right to left is 2, 4, 6 mg L^−1^.

The flocculation efficiency of the flocculation system increased with increasing chitosan concentration until the saturating concentration of 4 mg L^−1^ was achieved (Figure 7). At this concentration, the flocculation achieved a saturation point, and a further increase in chitosan concentration did not result in an increase of flocculation efficiency. To verify these hypotheses, chitosan concentrations of 2, 4, and 6 mg L^−1^ were selected for microscopic observation under 40 times magnification (Figure 8). The addition of chitosan flocculant results in the clustering of the cyanobacterial cells. The flocculation mechanism of chitosan is based on electric dipole neutralisation, where a positively charged polymer forms an ionic bridge between the negatively charged cells, resulting in agglomeration and settlement of the bacteria cells.

## 3. Discussion

The internally-illuminated photobioreactor operating in three light regimes allowed for an increase of the specific growth rate to 0.160 d^−1,^ significantly above the unoptimised lighting condition approach. There is a scarcity of similar studies that focus on the cultivation of thermophilic cyanobacteria on a bioreactor scale, especially at volumes higher than a couple of litres. Earlier work has shown growth rates of the *Thermosynechococcus* BP-1 strain in a 2.6 L photobioreactor at 0.130 d^−1^ [30]. Our previous studies have measured the growth rate of the E542 strain grown in simulated flue gasses using small-scale tubular bioreactors at 0.367 d^−1^ [5]. More optimised setups that utilised a 6 L flat-panel airlift photobioreactor equipped with flow-directing static mixers and downcomer domains allowed for much higher biomass productivities of 2.9 g L^−1^d^−1^. Whilst the report lacks exact calculations of doubling times, these could be estimated from the growth curves as 2.3 d^−1^ [31]. When it comes to another thermophilic strain, *Thermosynechococcus* CL−1 (TCL-1), which was cultivated in small 1–2 L photobioreactors, productivities exceeding those of the BP-1 strain of 3.5 g L^−1^d^−1^ have recently been achieved [32]. Another study on the 2.6 L helical photobioreactor for cultivation of a high-value product, C-phycocyanin, biosynthesized by (*Thermo*)*Synechococcus lividus* PCC6715 has achieved similar specific growth rates of 2.054 d^−1^ [33]. All these findings indicate that, to date, high productivities in thermophilic cyanobacteria are typically achieved in relatively small volume, high-complexity photobioreactors that require major investment costs. The present study is, to the best of our knowledge, the largest scale-up in the cultivation of thermophilic cyanobacteria reported to date.

Chitosan harvesting is relatively well-described among eukaryotic microalgae, but similar studies are less abundant for cyanobacteria. The utilisation of chitosan for harvesting has not been previously attempted for thermophilic cyanobacteria, but some conclusions could be drawn from related mesophilic strains. In this study, the obtained effective chitosan concentration that enabled effective harvesting of 90% of the biomass, i.e., 4 to 6 mg L^−1^ during 2 h of treatment, was at least one order of magnitude better than results obtained by most studies. These results could be translated to the utilisation of 2 mg of chitosan being capable of flocculating one gram of thermophilic cyanobacteria. In other studies, 250 mg of chitosan per g of stationary culture *Synechocystis* was required to achieve over 90% flocculation efficiency [34]. Another study on the same strain revealed that for flocculation efficiency exceeding 90% to be achieved within 1 h of settling requires a final chitosan concentration of at least 5 mg L^−1^ [35]. Interestingly, 90% flocculation efficiency in 1 h has also been achieved for the marine *strain Synechococcus elongatus* BDU 130192 when treated with a 16 mg L^−1^ final concentration of chitosan. Meanwhile, for the model marine strain *Synechococcus* PCC7002, less than 40% flocculation efficiency could be obtained under similar conditions [36]. When utilising chitosan to flocculate a filamentous strain *Pseudoanabaena*, over 90% efficiencies have been achieved with approximately 80 mg L^−1^ chitosan concentration [37]. Combinations of these findings suggest that chitosan-based harvesting is the most promising method for harvesting *Thermosynechococcus* E542.

## 4. Materials and Methods

### 4.1. Strain Cultivation

The thermophilic unicellular cyanobacterium *Thermosynechococcus elongatus* PKUAC-SCTE542 (E542 throughout the manuscript, equivalent to FACHB-2455) was originally isolated from the hot spring area of Ganzi Prefecture, Sichuan Province, China [38] and identified and described by Tang et al. [39] and developed into a microbial cell factory as described by Liang [5]. In this study, the strain was precultured as described by Liang et al. [5]. In short, the strain was grown in standard BG-11 medium pH 7.1 in 500 mL Erlenmeyer flasks at 45 °C, 45 µmol m^−2^ s^−1^, and 60 rpm until it reached the late exponential phase (*OD*_685_ 1.5). The precultures were transferred to the photobioreactor at 10% of its volume. The optical density at 685 nm was measured using an Epoch 96-well microplate reader; measurements were made in triplicates, and standard deviations of these measurements were calculated. Simultaneously, the growth curve was correlated to the dry weight of the biomass using a standard curve. The specific growth rates in the exponential growth phase µ [d^−1^] were determined with biomass concentrations represented as g L^−1^ to the Equation (1). The dry weights have been obtained using a standard curve that relates the optical density to the dry weight using an experimentally determined coefficient of 0.511 multiplied by the optical density [5]:

(1) $$\mu =\frac{lnX_{2}-lnX_{1}}{t_{2}-t_{1}}$$

where; *X*_1_ and *X*_2_ are dry weight biomass concentrations at times *t*_1_ and *t*_2_, respectively

The doubling times have been calculated from the specific growth rate using the following formula, where *μ* is the specific growth rate calculated with Equation (1):

(2) $$\mathrm{doubling}\;\mathrm{time}=\frac{\log\left(2\right)}{\log\left(1+\mu \right)}$$

### 4.2. Estimation of Light Attenuation

The light attenuation curve of *Thermosynechococcus* E542 has been studied using four hollow acrylic tubes with the same diameter and different heights (2, 4, 6, 10 cm) wrapped with opaque tin foil around the tube walls to minimise losses (Figure S1). Nine initial optical densities were tested (*OD*_685_: 0.04, 0.09, 0.15, 0.23, 0.44, 0.68, 0.83, 1.15, 1.47), at an incident light intensity *I*_0_ = 394.67 μmol m^2^ s^−1^.The residual photosynthetic photon flux density was measured for all conditions tested using the Handheld Spectrometer Lighting Passport APL-01 (Asensetek, Canada). Based on these results, the relationship was plotted (Figure 1), and light attenuation models were proposed. Mathematica 12.0 was used to calculate the parameters of the light distribution model *P_m_* (maximum light absorption coefficient), *K_Xc_* (scattering coefficient of light energy by bacterial cells) and *K_r_* (scattering coefficient of light energy by light range). The light distribution model in the built-in light source photobioreactor was obtained, and the light distribution curves at different concentrations and light ranges were plotted. OriginPro2018 was used to find respective correlations.

### 4.3. Light Attenuation Models

#### 4.3.1. Lambert–Beer Model

The light attenuation in the culture system was evaluated using the transformation of the Lambert–Beer equation:

(3) $$A\left(X\right)=\ln\left(\frac{I_{0}}{I}\right)=K_{a}XL$$

where *X* is the biomass concentration, *I*_0_ is the incident radiation intensity (120 μmol/m^2^ s^−1^), *I* is the transmitted radiation intensity, *L* is the optical length, and *K*_a_ is the attenuation coefficient at a given wavelength.

#### 4.3.2. The Hyperbolic Model

The hyperbolic model is defined by the following equation:

(4) $$A\left(X\right)=A_{max}X/(K_{at}X+X)$$

where *A*_max_ is the maximum optical attenuation coefficient, *K_at_* is the constant and *X* is the cell concentration.

### 4.4. Numerical Simulation of the Model

The Lambert–Beer model and hyperbolic model are universal models that may not accurately reflect the light distribution in an actual bioreactor. Therefore, the light distribution model of the bioreactor with an internal light source was used to simulate and solve the parameters:

(5) $$\ln(I_{0}/I)=\frac{P_{m}\cdot OD_{685\mathrm{nm}}\cdot L}{\left(K_{x_{c}}+OD_{685\mathrm{nm}}\right)\left(K_{r}+L\right)}$$

where *L* is the distance (cm) from a point inside the reactor from the reactor shell, *P_m_* represents the maximum light absorption coefficient, *K_xc_* represents the scattering coefficient of light energy by bacterial cells, and *K_r_* represents the scattering coefficient of light energy by optical path.

The data forms ln(*I*_0_*/I)*, *L* and *OD*_685nm_ were constructed in the *Mathematica* 12.0 program and iterated over the optical attenuation data for matrix calculation; nonlinear regression analysis was performed to fit the data to the model to obtain the parameters *P_m_* = 23.5558, *K_xc_* = 4.76169 and *K_r_* = 0.672164, *R*^2^ = 0.91. The light distribution model formula is as follows in Equation (4), while the light distribution curve is shown in Figure 3:

(6) $$\ln(I_{0}/I)=\frac{23.5558\cdot OD_{685\mathrm{nm}}\cdot L}{\left(4.76169+OD_{685\mathrm{nm}}\right)\left(0.672164+L\right)}$$

### 4.5. PBR Device Construction and Operation

#### 4.5.1. Materials

The internally-illuminated, cylindrical photobioreactor was constructed according to the scheme presented in Figure 4 using materials summarised in Table 4.

#### 4.5.2. Cultivation of *Thermosynechococcus* E542 in the Internally-illuminated PBR

The cultivation of *Thermosynechococcus* E542 in the internally-illuminated photobioreactor was performed in a thermostated growth chamber (Shanghai Yiheng Technical Co.) without external illumination. The temperature was set at 45 °C, and the culture was grown under continuous illumination. The following light intensity regime was applied: *OD*_685_ 0.1 to 0.2, 200 μmol m^2^ s^−1^; *OD*_685_ 0.2 to 0.4, 400 μmol m^2^ s^−1^; and, for *OD*_685_ > 0.4, 530 μmol m^2^ s^−1^. The strain was cultured in BG-11 medium supplemented with 0.1 M NaHCO_3_, with an initial *pH* of 8.65, and constantly aerated at the flowrate of 20 L min^−1^.

### 4.6. Preparation of the Flocculant and Measurement of Flocculation Efficiency

Biotechnology-grade chitosan (degree of deacetylation > 95%, viscosity 100–200 mPa.s) was purchased from Macklin Biochemical Technology Co., Ltd. (Shanghai, China) and dissolved in 1% dilute acetic acid at room temperature (25 °C) and stirred for 15 min. Five chitosan stock solutions (5, 10, 20, 40, 60 mg L^−1^) of different mass concentrations of the flocculant were prepared, whilst 1% dilute acetic acid, and deionised water were used as controls. Flocculation efficiency was studied at room temperature (25 °C). Briefly, 1 mL of chitosan solution at different concentrations was added to the 15 mL centrifugal tube containing 9 mL of cyanobacterial culture at the *OD*_685_ 1.5 corresponding to the dry weight of approximately 2 g L^−1^, resulting in final chitosan concentrations of 0.5, 1, 2, 4, 6 mg L^−1^. The solution was mixed to homogeneity, and the optical density *OD*_685_ of the culture was measured at the level of 5 cm from the top of the centrifugal tube at the following time intervals: 0, 20, 40, 60, 90, 120 min. All these measurements were performed in triplicates, and their standard deviations were calculated. The flocculation efficiency was calculated using the following equation:

(7) $$R=\frac{OD_{O}-OD_{f}}{OD_{O}}\times 100\%$$

where *R* is the flocculation efficiency, *OD_O_* is the optical density at time 0, and *OD_f_* is the optical density after flocculation time is complete. After 120 min of flocculation experiment, the bottom layer of the centrifugal tube was analysed with light microscopy (Olympus BX53).

## 5. Conclusions

The present study explored the large-scale cultivation of *Thermosynechococcus* E542 in a 40-litre photobioreactor. The photobioreactor was operated with a light regime derived from a light attenuation study. The light attenuation characteristics of the cyanobacterial cultivation were fitted with the hyperbolic model. Among two tested illumination methods, internal and external, the former was significantly superior, resulting in a two-fold better growth rate and four-fold higher biomass concentration. This difference can be attributed to the light limitation of the externally-illuminated photobioreactor. During the large-scale cultivation process, it was also shown that thermophilic cyanobacteria should be supplemented with bicarbonate to avoid carbon limitation. Finally, it was shown that chitosan-based harvesting of the thermophilic cyanobacteria is a very promising route to address the problems associated with biomass collection of unicellular cyanobacteria. This study has shown that concentrations of chitosan as low as 4 mg L^−1^ are sufficient for the harvesting of *Thermosynechococcus* E542. The study indicates potential methods of increasing biomass productivity by appropriately designing the bioreactor and modulating the light supply to the culture; furthermore, this study suggests effective methods of biomass harvesting. The proposed solutions can have a positive impact on the development of effective methods of cyanobacterial biomass cultivation and processing.

## Figures and Tables

**Table 1:** Fitting formula and correlation test of light attenuation.

Model: ExpDec1	I = *A*1 *exp*(−*r/t*_1_) + *y*_0_	
*OD*_685nm_	Fitting Formula	*R* ^2^
0.04	*I* = 292.78*exp*(−*r/*1.92) + 99.07	0.96788
0.09	*I* = 338.93*exp*(−*r/*0.98) + 55.29	0.97105
0.15	*I* = 368.38*exp*(−*r/*0.95) + 26.03	0.99044
0.23	*I* = 380.67*exp*(−*r/*0.78) + 13.93	0.9955
0.44	*I* = 391.42*exp*(−*r/*0.67) + 3.24	0.99963
0.68	*I* = 393.31*exp*(−*r/*0.56) + 1.36	0.99993
0.83	*I* = 394.07*exp*(−*r/*0.48) + 0.60	0.99999
1.15	*I* = 394.41*exp*(−*r/*0.42) + 0.26	1
1.47	*I* = 394.58*exp*(−*r/*0.35) + 0.09	1

Note: the function *y* = *A*1 *exp*(−*x/t*_1_) + *y*_0_ defines the general exponential function that describes the relationship between parameters of light intensity and optical length; the curve fitting was performed to solve three parameters *A*1, *y*_0_ and *t*_1_ at *r* is the optical length.

**Table 2:** Components and dimensions of the bioreactor.

No.	Component	Parameters	
(1)	Diffuser stone	*d*_max_ = 25 cm	*h* = 15 cm
(2)	Harvesting valve	*d*_1_ = 1.5 cm	*-*
(3)	Reactor	*V* = 40 L	*h* = 100 cm
(4)	Glass tube	*d*_2_ = 5 cm	*h* = 80 cm
(5)	LED	24 V	14 w/m
(6)	Air pump	0.045 Mpa	50 L/min
(7)	Reactor cover	*d* = 30 cm	*h* = 2 cm
(8)	Power supply	Voltage range 12–24 V	Output current 1.5–6.5 A

**Table 3:** The ratio of light area to dark area in the bioreactor. Where: *r_s_* is the light area radius; *R-r_s_* is the dark area radius; *V_L_:V_D_* is the light area and dark area ratio; and *V_D_/V* is the ratio of light area compared to the whole area of reactor.

*OD*_685nm_									
	0.04	0.09	0.15	0.23	0.44	0.68	0.83	1.15	1.47
*r_s_*	5.06	3.18	2.62	2.45	2.32	2.30	2.29	2.29	2.29
*R-r_s_*	9.94	11.82	12.38	12.55	12.68	12.70	12.71	12.71	12.71
*V_L_:V_D_*	1:2	1:4	1:5	1:5	1:5	1:6	1:6	1:6	1:6
*V_D_/V*	0.66	0.79	0.83	0.84	0.85	0.85	0.85	0.85	0.85

**Table 4:** Components of the internally illuminated photobioreactor.

	Component	Material	Supplier
(1)	Diffuser stone	Emery	Danies Aquarium, China
(2)	Harvesting valve	Acrylic	Chuang Yi, China
(3)	Reactor	Acrylic	Chuang Yi, China
(4)	Glass tube	Glass	Hua Xing, China
(5)	LED	LED	Kaloulight, China
(6)	Air pump	-	Belos, China
(7)	Reactor cover	Acrylic	Chuang Yi, China
(8)	Power supply	-	Bao an Qua Hao, China

## Data Availability

Data is contained within the article and Supplementary Material.

## Supplementary Materials

The following are available online at https://www.mdpi.com/article/10.3390/plants10081540/s1, Figure S1. Light attenuation measurement of cyanobacteria E542. The figure presents the light intensity measurements through different length of a cyanobacterial culture column using a handheld photometer (APL-01).
