# Supplementary figures and images for: Debottlenecking Thermophilic Cyanobacteria Cultivation and Harvesting through the Application of Inner-Light Photobioreactor and Chitosan
Source: Plants (Basel). 2021 Jul 27;10(8):1540. doi: 10.3390/plants10081540 (PMC8400073; doi:10.3390/plants10081540)

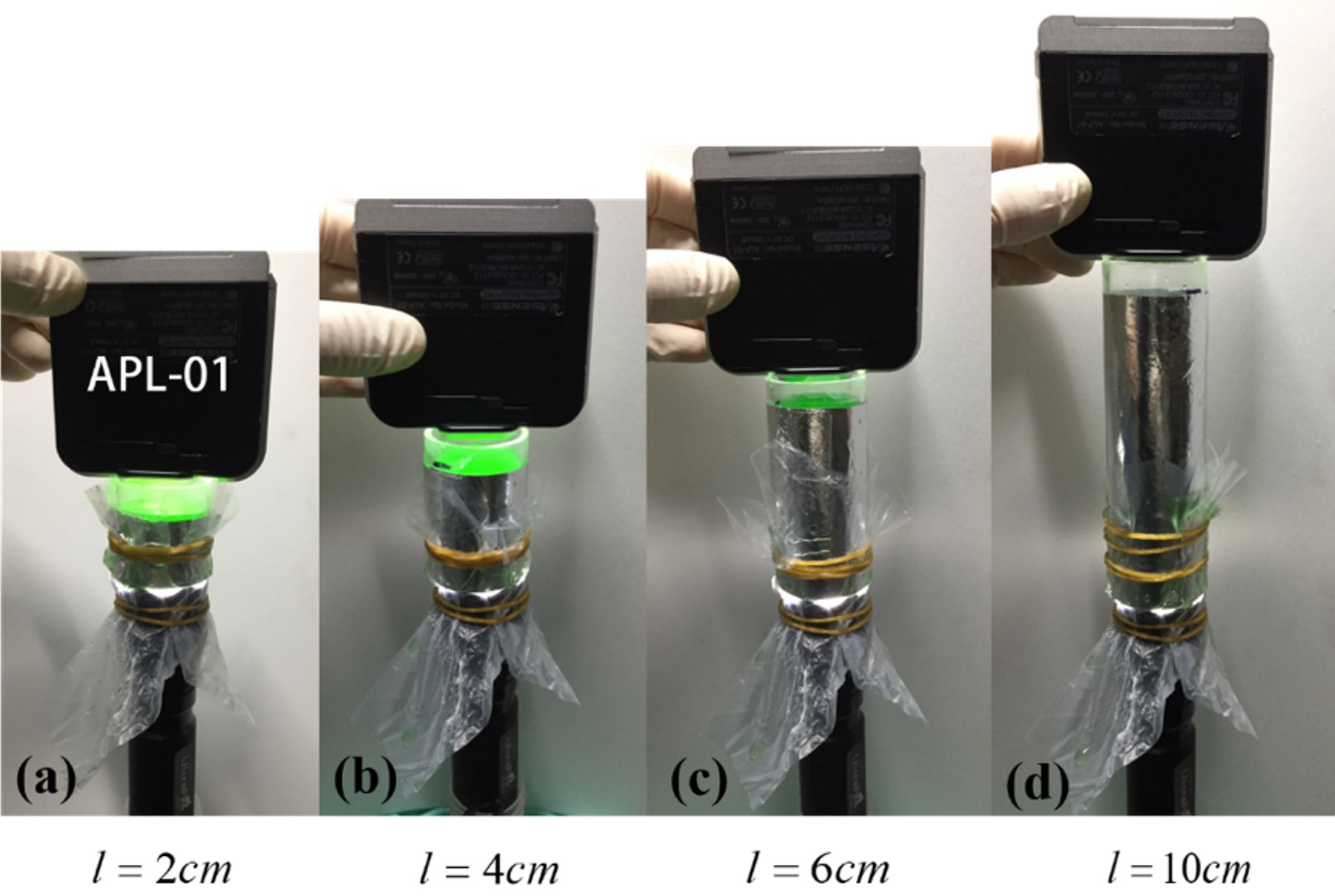

Supplement: Supplementary file 1 [file plants-10-01540-s001.zip › plants-1307305-supplementary.JPG]
